# Revealing Unconscious Consumer Reactions to Advertisements That Include Visual Metaphors. A Neurophysiological Experiment

**DOI:** 10.3389/fpsyg.2020.00760

**Published:** 2020-05-12

**Authors:** Jesús García-Madariaga, Ingrit Moya, Nuria Recuero, María-Francisca Blasco

**Affiliations:** Management and Marketing Department, Complutense University of Madrid, Madrid, Spain

**Keywords:** consumer neuroscience, cognitive load, visual metaphors, advertising, attitude toward the ad, electroencephalogram, eye tracking, galvanic skin response

## Abstract

The main challenge of advertising is to catch consumers’ attention and evoke in them positive attitudes to consequently achieve product preference and higher purchase intentions. In modern advertising, visual metaphors are widely used due to their effects such as improving advertising recall, enhancing persuasiveness, and generating consumers’ positive attitudes. Previous research has pointed out the existence of an “inverted U-curve” that describes a positive relationship between the conceptual complexity of metaphors and consumers’ positive reactions to them, which ends where complexity outweighs comprehension. Despite the dominance of visual metaphors in modern advertising, academic research on this topic has been relatively sparse. The inverted U-curve pattern has been validated regarding ad appreciation, ad liking, and purchase intention by using declarative methods. However, at present, there is no evidence of consumers’ neurophysiological responses to visual metaphors included in advertising. Given this gap, the aim of this research is to assess consumer neurophysiological responses to print advertisements that include visual metaphors, using neuroscience-based techniques. Forty-three participants (22W–21M) were exposed to 28 stimuli according to three levels of visual complexity, while their reactions were recorded with an electroencephalogram (EEG), eye tracking (ET), and galvanic skin response (GSR). The results indicated that, regardless of metaphor type, ads with metaphors evoke more positive reactions than non-metaphor ads. EEG results revealed a positive relationship between cognitive load and conceptual complexity that is not mediated by comprehension. This suggests that the cognitive load index could be a suitable indicator of complexity, as it reflects the amount of cognitive resources needed to process stimuli. ET results showed significant differences in the time dedicated to exploring the ads; however, comprehension doesn’t mediate this relationship. Moreover, no cognitive load was detected from GSR. ET and GSR results suggest that neither methodology is a suitable measure of cognitive load in the case of visual metaphors. Instead, it seems that they are more related to the attention and/or emotion devoted to the stimuli. Our empirical analysis reveals the importance of using neurophysiological measures to analyze the appropriate use of visual metaphors and to find out how to maximize their impact on advertising effectiveness.

## Introduction

Marketing scholars and practitioners are continuously facing the challenge to find out how to enhance advertising effectiveness. The decrease of traditional advertising media such as TV and newspapers, the rise of new ones like mobiles or videos, and the growth of interactive and targeted advertising represent a huge limitation to print advertising, characterized by a static image. Given this limitation, graphic print advertising must focus on seeking the most optimal design to catch consumers’ attention and evoke positive attitudes, in order to trigger a higher preference for the products and, consequently, higher purchase intentions.

Advertisers and academics have analyzed the key factors that influence effective print advertisement such as element location (Garcia et al., 2000; Girisken and Bulut, 2014), advertisement size (Pieters and Wedel, 2004), images (Gakhal and Senior, 2008; Cook et al., 2011; Bastiaansen et al., 2016; Tomaselli Fidelis et al., 2017), exposure duration (Elsen et al., 2016) and messages (Thomsen and Fulton, 2007). However, the complexity of visual images and their impact on print advertising have been poorly studied until Phillips and McQuarrie’s (2004) research, which provides a significant theoretical review on aspects of visual complexity by analyzing visual rhetoric in advertising.

Images themselves can be extremely complex, as they are “capable of representing concepts, abstractions, actions, metaphors and modifiers” (Scott, 1994, p. 253). A single image can contain many sophisticated interrelated signs and multiple meanings for viewers or readers (Bulmer and Buchanan-Oliver, 2006). Therefore, as Phillips and McQuarrie (2004) suggest, the role of images in advertising needs in-depth study, as they are not necessarily analogous to visual perception but are rather symbolic artifacts.

A rhetorical figure is an artful deviation relative to audience expectation (McQuarrie and Mick, 1996), which can comprise a variety of different forms such as rhyme, antithesis, ellipsis, metaphor, and pun. Rhetorical figures have been cataloged and studied primarily from a text perspective, although literature provides evidence that the artful deviation characteristic of figures also can be constructed out of pictorial elements in advertising (Forceville, 1994, 1996, 2005; McQuarrie and Mick, 1996; Foss, 2005; Mohanty and Ratneshwar, 2014).

Among the visual rhetoric figures, metaphors are the most commonly used because, according to the theory, they can formulate, sustain, or modify the attention, perceptions, attitudes, or behaviors of their audiences (Foss, 2005); they also provide a novel way of communicating product attributes to consumers, and they can enhance ad recall and produce more positive attitudes (McQuarrie and Mick, 2003; Norris et al., 2012).

Scholar research defines metaphors as comparisons between two things that are originally different in nature but have something in common (van Mulken et al., 2014), and where one concept is understood in terms of another (Peterson, 2019). Among the visual rhetoric literature, there are three main approaches to classify visual metaphors (Forceville, 1996, 2005, 2008; Phillips and McQuarrie, 2004, 2009; Gkiouzepas and Hogg, 2011), and although each author uses different terms to name them, it is possible to distinguish three types of metaphors: (1) a comparison based on two items that are presented separately (i.e., similes or juxtaposition); (2) a combination of two things that evoke a single concept (i.e., hybrid metaphor, synthesis, or fusion); and (3) an absent object that is evoked by an image (i.e., contextual image or replacement). All authors indicate an increasing degree of complexity going from no metaphor to juxtaposition, to fusion, and finally to replacement.

Consumer studies have concluded that advertisements with complex layouts evoke positive attitudes (McQuarrie and Mick, 1996, 2003; van Mulken et al., 2010, 2014), high appreciation (van Mulken et al., 2010), advertisement recognition (McQuarrie and Mick, 1996, 2003; Norris et al., 2012), and purchase intentions among consumers (Jeong, 2008; van Hooft et al., 2013; Myers and Jung, 2019). However, the evidences provided by these researches is all based on results from declarative studies. Thus, at present, there are no studies developed using other research methodologies. This lack of evidence of non-declarative reactions motivates the present research, which seeks to fill this gap by investigating experimentally the neurophysiological responses of consumers to visual metaphors included in advertising by applying neuromarketing techniques.

## Conceptual Tenets and Literature Review

### Visual Complexity in Advertising

As mentioned, previous studies have consistently reported that advertisements with complex layouts result in audiences’ more positive attitudes than advertisements based on stand-alone images (Phillips, 2000; Jeong, 2008; Pieters et al., 2010; van Mulken et al., 2010, 2014). Once a subject resolves the riddle, a positive attitude toward the advertisement emerges that consequently yields a significant improvement of ad recall, brand recognition (McQuarrie and Mick, 2003; Norrick, 2003), product perception (McQuarrie and Mick, 2009), and purchase intentions (Ang and Lim, 2006; Jeong, 2008; van Hooft et al., 2013; Myers and Jung, 2019).

It seems that decoding the message increases the subject’s sense of pleasure and decreases the sense of tension, leading to the enhancement of the subject’s attitude toward the ad (Aad) (Jeong, 2008) and to improve the ad persuasiveness (Burgers et al., 2015). In this respect, Hornikx and le Pair (2017) determined that a positive Aad occurs when consumers are exposed to advertisements that require higher cognitive effort than when they are presented with advertisements that do not require much cognitive effort.

According to Phillips and McQuarrie (2004), there are two determinant factors for the processing of visual rhetoric figures: the richness of the figure and its complexity. Putting those two dimensions together, visual metaphors can vary from simple and readily interpretable figures to highly complex figures open to a wide range of interpretations. Thus, excessively complex metaphors may fail to be comprehended and, consequently, cease having a positive impact (Phillips and McQuarrie, 2004). This effect is related to Berlyne’s (1971) theory, which suggests that the relationship between complexity and pleasure could be explained by an inverted U-curve whose tipping point is reached when complexity outweighs comprehension (van Mulken et al., 2014).

The pleasure evoked by complex visual images used in advertising has been studied from different perspectives. Previous studies suggest that if metaphors demand too much or too little cognitive processing effort, consumers may opt out, and appreciation will decrease; thus, advertisement appreciation follows the pattern of the aforementioned inverted U-curve (Phillips, 2000; McQuarrie and Mick, 2003). In the same line, van Mulken et al. (2010, 2014) validated the inverted U-curve pattern in advertisement appreciation and pointed out that visual metaphors of moderate complexity are the most effective. Moreover, van Hooft et al. (2013) studied the inverted U-curve as a function of liking and purchase intention. They confirmed the pattern regarding preference but found only partial confirmation regarding purchase intention because, although they found that more complex metaphors lead to lower purchase intentions, there was no difference between juxtapositions and fusions regarding this variable.

Despite of the valuable findings of the aforementioned research, empirical evidence for the inverted U-curve is still relatively scarce, and its validity has not yet been proven regarding Aad, a concept extensively examined that reveals consumers’ precise perceptions and impressions toward advertisement designs (Huhmann and Limbu, 2016). Neither it has been proven on preference. Based on this lack of evidence, we will validate the presence of the inverted U-curve pattern in those two important indicators of advertising effectiveness: Aad and preference. Besides, due to the importance of purchase intentions, we will also include it in order to find if the pattern could be validated in different product categories than that used by van Hooft et al., 2013. Hence, it is postulated:

Hypothesis 1: *The effects of metaphors on* (*a*) *Aad*, (*b*) *purchase intention, and* (*c*) *preference follow the inverted U-curve pattern according to which there is a positive relationship between complexity and positive feelings until a tipping point is reached where complexity exceeds comprehension.*

### Processing of Visual Metaphors

According to Phillips (2003), the usage of metaphors in advertising mainly impacts four variables: attention, elaboration, pleasure, and liking. As visual metaphors are defined as artful deviations from expectations (McQuarrie and Mick, 1999), they give rise to incongruity that certainly attracts attention and prompts exploratory behaviors (Kaplan, 1990; Jeong, 2008; Mohanty and Ratneshwar, 2014). Once attention is caught, the consumer is forced to decipher the underlying message. This means that attention is retained and that the consumer must devote some time to provide a meaning for the ad and to elaborate the message. Finally, the extra effort is rewarded with the pleasure of having been able to solve the puzzle, and it leads to more positive attitudes toward the advertisement (van Hooft et al., 2013).

In cognitive psychological terms, elaboration “indicates the amount, complexity, or range of cognitive activity occasioned by a stimulus” (McQuarrie and Mick, 1999. p 39). When the viewer draws an inference or generates assumptions and integrates them with his/her prior knowledge, this launches an elaboration process where working memory is increasingly taxed to the extent that complexity increases (Peterson, 2019).

The increased elaboration of visual metaphors has been proven in previous studies (McQuarrie and Mick, 1999, 2003; Phillips, 2000**;** Jeong, 2008**;** Chang and Yen, 2013**)**, where it is stated that more complex visual figures lead to more cognitive elaboration. Such higher elaboration is a consequence of comprehension efforts, and it manifests as an enhanced memory of the ad (Phillips and McQuarrie, 2004).

Those previous findings have been very valuable for marketing, however, their weakness lies in the fact that they are derived from declarative methodologies that are inevitably biased by subjective considerations (Hsu, 2017). To overcome this situation, in the last few years, neurophysiological techniques have begun to be applied, mainly because of their ability to provide additional insights crucial to understanding consumers’ behavior (Dimofte, 2010; Gattol et al., 2011).

Due to its recent adoption in marketing, research on visual metaphors in advertising by applying neuroscientific techniques is still scarce (Bambini et al., 2016). The studies on this matter are mainly focused on the analysis of textual metaphors and are mostly restricted to semantic processing (Sotillo et al., 2004; Lachaud, 2013; Rataj, 2014).

In spite of that, we note that studies made with an electroencephalogram (EEG), and especially those based on the recording of event-related brain potentials (ERPs)–an EEG methodology that offers great insights into processing mechanisms with millisecond precision (Kappenman and Luck, 2012)–suggest a biphasic pattern of brain activity, with earlier negativity (N400) followed by later positivity (P600/LPC) (Coulson and Van Petten, 2002; Weiland et al., 2014; Bambini et al., 2016). N400 is linked to efforts in terms of lexical access and semantic representation (Kutas and Federmeier, 2011), whereas P600 is usually observed for syntactic operations (Brouwer et al., 2012).

In the same line, Pileliene and Grigaliunaite (2016) analyzed the allocation of attentional resources to process advertising with complex layouts (not metaphorical), through the use of P300, a component that provides information about the neural activity of cognitive operations (Ma et al., 2008). The obtained results revealed that a complex layout in an advertisement leads to more attentional and cognitive resources being engaged in processing the advertisement as well as to the higher emotional value to consumers.

Moreover, regarding temporal and spatial studies of the brain, the EEG study developed by Cardillo et al. (2012) found prefrontal and left posterior temporal activations in the presence of higher cognitive processes of comparison and categorization related to the elaboration of metaphorical contents. These findings are consistent with those of Anderson et al. (2011), who studied cognitive load across multiple visualization types and found that, as Klimesch (1999) suggested, EEG oscillations in the alpha band reflect cognitive performance and that, in particular, the movement of the individual alpha frequency outside of the 8–12 Hz band of frequencies may indicate a cognitive overload induced by a too-complex visualization task.

On the other hand, studies using eye tracking (ET) revealed that the time devoted to exploring the stimuli could be indicative of the cognitive processes involved in comprehending the metaphors, as experimental studies showed that extra time is needed to comprehend more complex metaphors (Raney et al., 2014). Besides, the implication of more visual attention when more complex advertisements are presented could be translated into longer ET fixation time (Pieters et al., 2010).

Finally, some authors have suggested that galvanic skin response (GSR) seems to be a suitable tool for measuring cognitive activity and have pointed out the correlation between GSR features and cognitive functions and more specific cognitive workload (Miller and Shmavonian, 1965; McEwen and Sapolsky, 1995; Shi et al., 2007; Nourbakhsh et al., 2012).

Based on the preceding discussion, we can identify a positive relationship between complexity and elaboration that can be represented as a higher cognitive load as metaphors increase their complexity. According to literature, the cognitive load can be measured with EEG, analyzing oscillations of alpha–theta bands; with ET, analyzing the time devoted to exploring the visual metaphor; and by analyzing GSR features. Hence, it is hypothesized:

Hypothesis 2: *To the extent that a visual metaphor increases its difficulty, the subject will have a higher cognitive load. This situation will be reflected in* (*a*) *a longer time to explore the advertisement*, (*b*) *a higher index of EEG cognitive load, and* (*c*) *a higher index on GSR activation.*

## Materials and Methods

### Participants

Forty-three undergraduate students (22 women, 21 men) voluntarily participated in the study in June 2018. The mean age was 23.3 years with a standard deviation of 2.8 years. The participants were recruited using convenience sampling. All participants were right-handed, healthy people with normal or corrected-to-normal vision and were free of any hearing problems. All participants provided signed consent before participating and received monetary compensation at the end of the session.

### Measurements

#### Consumer Neuroscience Techniques

##### Electroencephalograph

The EEG is a measurement of the whole sphere of brainwave activity emerging in various cortical areas, which helps to understand the way the brain responds to various stimuli. EEG is a non-invasive instrument that provides information from areas underneath the cortex and, combined with other instruments, may provide very accurate results on a subject’s response to a marketing stimulus (Du Plessis et al., 2011).

Cerebral activity was recorded using the Bitbrain Versatile EEG with 16 channels at a sampling rate of 256 Hz, while impedances were kept below 5 kΩ. For the experiment, we used 12 electrodes placed by following the International 10–20 system.

##### Eye tracking

This biometric technique is based on the relationship between human eye movements, visual attention, and information acquisition, with the latter two both being closely related to higher-order cognitive processes (Ares et al., 2014). ET has a high temporal resolution (60–120 Hz) and uses an optical camera to identify the position of the pupil and cornea using near-infrared light pointed at the cornea and reflected off it (Venkatraman et al., 2015). When the eye moves across a spatial stimulus, the difference between the incoming and outgoing angle of the infrared light beam changes, indicating the specific position on the stimulus to which the eye moves (Pieters and Wedel, 2004).

Consumers’ behavior is measured with an ET technique by recording either the number of fixations or dwell time of the eyes during an individual or group exposure to external stimuli. The specific ET device used in the present study was a Tobii X2-30 Eye-Tracker Compact Edition, a screen-based eye tracker capturing gaze data at 60 Hz.

##### Galvanic skin response

The GSR is defined as a change in the electro-physiological properties of the skin due to sweat gland function. GSR provides an indication of changes in the human sympathetic nervous system (SNS) (Shi et al., 2007) and is well known as a robust and easily captured physiological tool available at low cost (Nourbakhsh et al., 2013). The GSR measures the electrodermal response that occurs when the skin becomes a better electrical conductor due to increased activity of the sweat glands because of the exposure to a specific stimulus (Potter and Bolls, 2012). Therefore, the skin conductance amplitude provides a direct measure of subjects’ arousal (Venkatraman et al., 2015). The GSR device used in the present study to get the arousal was the Bitbrain GSR ring, a wireless device for real-time monitoring of electrodermal and cardiac activity.

#### Neurophysiological Measurements

Neurophysiological measurements comprise cognitive load, time in AOI and arousal. These measures are described following and the instruments used to measure them are related in Table 1.

**TABLE 1:** Summary of Consumer neuroscience measures.

Consumer neuroscience measures	Instrument
Time to AOI	ET
Cognitive load	EEG
Activation	GSR

*AOI, area of interest; ET, eye tracking; EEG, electroencephalogram; GSR, galvanic skin response.*

##### Cognitive load index

The EEG technique can be used to obtain many different psychological metrics such as cognitive workload. Studies on the area have found that EEG power in the theta and alpha frequency range is related to cognitive performance (Antonenko et al., 2010). In fact, with increasing task demands, theta synchronizes (increases), whereas alpha desynchronizes (decreases). That is the situation of visual attention and semantic tasks, primary factors that lead to a suppression (decrease) of the alpha rhythm in the prefrontal cortex (Klimesch, 1999, 2012; Gevins and Smith, 2003; Anderson et al., 2011).

Based on previous studies (Klimesch, 1999, 2012; Gevins and Smith, 2003; Antonenko et al., 2010; Anderson et al., 2011), in the present study, the cognitive load was calculated by computing the ratio between power in the theta band in frontal channels (F3, F4) and power in the alpha band in parietal channels (P3, P4).

##### Time spent exploring stimuli (Time in Area of Interest)

The ET technique has been used as a direct measure of attention by analyzing the number of fixations on specific areas, the viewing time, or the time that users take to reach each area (Rebollar et al., 2015) and also as a measure of the cognitive processes involved in comprehension (Raney et al., 2014). ET and pupillary responses may also provide additional insights into the cognitive load concerning with respect to visualization studies (Anderson et al., 2011). ET has great potential for objectively assessing consumers’ perception of visual stimuli (Wedel and Pieters, 1989, 2008) and is being increasingly used in consumer science (Ares et al., 2013; Piqueras-Fiszman et al., 2013; Mitterer-Daltoé et al., 2014; Fenko et al., 2018).

Taking into account that there is evidence regarding “a sufficiently close connection between time spent fixating on display items and the amount of cognitive processing” (Graesser et al., 2005, p. 1237) that has been validated by previous studies (Lang et al., 2002; Graesser et al., 2005; Pieters et al., 2010; Lagerwerf et al., 2012; Raney et al., 2014), the present study employs the total time spent looking at each metaphor [time in area of interest (AOI)] as indicator of cognitive elaboration.

To get the time in AOI, we previously defined the AOIs on each image by selecting the area of the metaphor. In order to avoid the bias derived from having a different number of images according to the type of metaphor, we defined as “area of interest” the same zone (same size and shape) for all four conditions in each set.

##### Arousal

Previous studies have found that the GSR signal represents a suitable measure for detecting emotional responses but also for differentiating between stress and cognitive load (Shi et al., 2007; Nourbakhsh et al., 2013). Besides, Mühl et al. (2014) proved that GSR offers an unobtrusive and continuous measure sensitive to cognitive workload. Subjects’ arousal was obtained by computing the skin conductance response (SCR) amplitudes taking into account that phasic SCRs are a reliable concomitant of states of arousal (Benedek and Kaernbach, 2010a).

#### Declarative Questionnaire

A computer-based questionnaire was also applied, through an Internet platform, to obtain declarative Aad, purchase intention, preference, and perceived complexity (see Table 2).

**TABLE 2:** Summary of declarative measures.

Declarative measures	Instruments	
Attitude toward the ad α = 0.979	7-point Likert scale	Not interesting/very interesting, not appealing/very appealing, dislike/like, and bad/good
Purchase intention α = 0.972	7-point Likert scale	Not likely/very likely to buy and you are very likely to recommend this product/you are not likely to recommend this product
Preference	Ranked from 1 to 4	1 = the most preferred stimulus 4 = the least preferred stimulus
Perceived complexity α = 0.981	7-point Likert scale	Unclear/straightforward and difficult to understand/easy to understand

(1) Aad was measured adopting Lutz et al.’s (1983) and MacKenzie et al.’s (1986) measure, by using four semantic differential items: not interesting/very interesting, not appealing/very appealing, dislike/like, and bad/good. The response options were on a seven-point scale. Cronbach’s alpha for this construct was 0.979.
(2) Purchase intention was measured as in previous studies (Ang and Lim, 2006; Jeong, 2008; van Hooft et al., 2013), on a seven-point Likert scale that comprised the next two semantic differential items: not likely/very likely to buy and you are very likely to recommend this product/you are not likely to recommend this product. Cronbach’s alpha for this construct was: 0.972.
(3) Participants ranked their preference from 1 (the most preferred stimulus) to 4 (the least preferred stimulus).
(4) Perceived complexity was measured as in van Mulken et al. (2010), on the basis that “more complex metaphors might on average be less well understood than less complex metaphors” (van Mulken et al., 2010, p. 3425). This item was measured on a seven-point Likert scale that comprised the next two semantic differential items: unclear/straightforward and difficult to understand/easy to understand. Cronbach’s alpha for this construct was: 0.981.

### Stimuli

**FIGURE 1:**
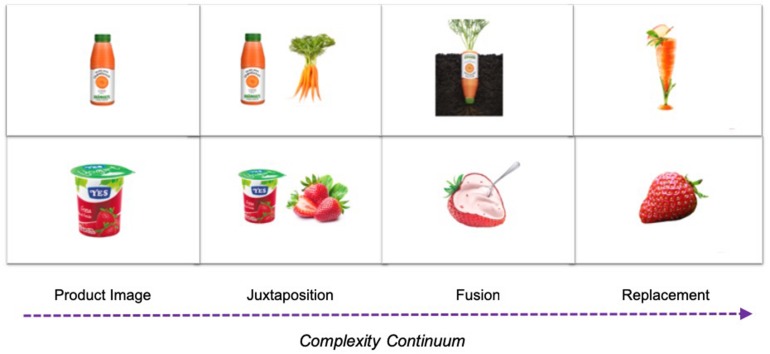
Example of sets of four print advertisements designed for the study.

A within-subjects research was conducted to assess consumer responses to print advertisements that include visual metaphors. Twenty-eight advertisements were developed in total, seven sets of four print advertisements for seven different product categories (see example in Figure 1). To build the metaphorical ads, the start point was a product image without any metaphorical content, also taken as the control condition. On the basis of that neutral point, we developed three metaphorical ads following the three main approaches to classify visual metaphors (Forceville, 1996, 2005, 2008; Phillips and McQuarrie, 2004, 2009; Gkiouzepas and Hogg, 2011) according to which there are three types of metaphors: (1) a comparison based on two items that are presented separately (i.e., similes or juxtaposition); (2) a combination of two things that evoke a single concept (i.e., hybrid metaphor, synthesis, or fusion); and (3) an absent object that is evoked by an image (i.e., contextual image or replacement).

Thus, we created seven sets of four images corresponding to three levels of complexity (juxtaposition, fusion, and replacement) and a control condition. Each set corresponded to one of the product categories (yogurt, juice, milk, tea, coffee, tomato sauce, and insecticide) selected on the basis of a focus group performed with eight people from 18 to 35 years, in which age range were found high levels of familiarity with categories of food, house, and personal care.

All images used were full-color, and product images were previously used in real printed or Internet ads. However, in order to avoid familiarization bias (Gregg and Klymowsky, 2013), although all brands and products used were real, they were not marketed in Spain.

### Procedure

#### Pre-test

Taking into account that metaphorical content could have multiple interpretations (Utsumi, 2007) a pre-test was conducted to assess the correct interpretation of metaphors to be used in the experiment. Sixty participants were invited to examine all the advertisements created and to answer whether they had recognized the concept expressed by each metaphor. To assess the metaphor comprehension, we applied the “valid/invalid” criteria of Morgan and Reichert (1999) according to which a valid interpretation of the metaphor is one in which the subject identified a relationship between the product and the concept used to create the comparison that is correctly supported by the metaphor. The obtained results showed that for each product category tested, more than 70% of participants made a correct identification of the metaphorical concept. We also checked that the complexity continuum for each set of images followed the described scale of complexity. It means that for each set of metaphors (juxtaposition, fusion, and replacement), there was an increase in complexity equal to that stated in literature.

#### Experimental Procedure

The study was performed at the Laboratory of Neuromarketing of Complutense University of Madrid, and its total duration was 60 min, including both blocks to be described in the following paragraphs. All 43 participants were right-handed, healthy people with normal or corrected-to-normal vision and were free of any hearing problems. All participants provided signed consent before participating and received monetary compensation at the end of the session.

The experimental procedure had two phases, which entailed the use of neurophysiological (Block 1) and declarative (Block 2) methods. In Block 1, after briefing the protocol to the participants, they were sat in front of the computer screen where the ET was installed and were affixed with the EEG and GSR devices for collecting their brain electrical activity and skin conductance. The screen used was 21 inches with full HD resolution (1,920 × 1,080 pixels).

To calibrate the ET, subjects were instructed to follow the points appearing on the screen with their sight without moving their heads. Once the ET was calibrated, the researcher checked that the signal of all three devices was good and started running the experiment. It is important to highlight that in order to measure the workload, the emotional activation, and the time spent exploring each ad, we used simultaneous EEG, GSR, and ET measurements during the whole experiment. The software used to present stimuli and simultaneously record data was SensLab, developed by Bitbrain.

**FIGURE 2:**
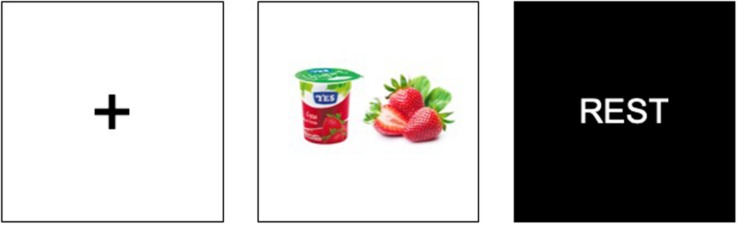
Stimuli presentation Block 1.

In this block, the 43 participants were exposed to the 28 aforementioned stimuli (7 product categories × 4 complexity modification designs), presented individually and randomly. First, a fixed cross was presented in the middle of the screen, followed by the stimulus for 5 s, and then a black slide, with the word “Rest,” so participants could take a 2 s rest (see Figure 2).

During Block 2, which entailed the declarative test phase, participants had to answer the questionnaire while they were visualizing the stimuli individually. The respondents initially answered the Aad scale for the 28 advertisements. The second scale, with the same structure, asked participants about their purchase intention. The third task was to rank the four advertisement modulations (product, fusion, juxtaposition, and replacement) from 1 to 4, to assess their preference. Finally, the last declarative section asked about their perceived complexity. In this second block, the exposure time depended on the subject’s response time instead of being standardized as in Block 1.

## Results

### Data Analysis

All raw data coming from the neurophysiological techniques were provided by SennsLab.

Raw EEG data were first filtered using a band-pass filter between 1 and 25 Hz with a four-order Butterworth filter. After that, a filtering pipeline was implemented. First, an ASR (artifact subspace reconstruction) filter was used to remove big amplitude artifacts (Mullen et al., 2013). Then ICA (independent component analysis) was performed in order to separate the EEG data info into independent components (Hyvarinen, 1999) to subsequently apply MARA, which is a machine learning–based algorithm that classifies automatically ICA components as artifacts or as clean data (Winkler et al., 2011). Once the signal was clean, we computed the cognitive load as the ratio between power in the theta band in frontal channels (F3, F4) divided by power in the alpha band in parietal channels (P3, P4) (Gevins et al., 1998; Klimesch, 1999; Gevins and Smith, 2003). To get the frequency bands, we first applied the Welch method to obtain the power spectral density. Theta and alpha bands were individualized using IAF (individualized alpha frequency) analysis (Doppelmayr et al., 1998).

Skin conductance data are usually characterized by a sequence of overlapping phasic SCRs overlying a tonic component (Benedek and Kaernbach, 2010b). The extraction of the skin conductance data followed three steps: (1) the deconvolution of recorded data and the subsequent estimation of (2) tonic and (3) phasic activity. After application of a low-pass filter to eliminate the muscle noise in order to detect more accurately the sweating peaks to the GSR signal, information on the subjects’ arousal was obtained by computing the SCR amplitudes taking into account that phasic SCRs are a reliable concomitant of states of arousal (Benedek and Kaernbach, 2010a).

The post-processed EEG and GSR signals, next to the ET information about time in AOI were subsequently analyzed by using SPSS. The declarative information also was analyzed using that statistical software.

The data analysis was performed in three stages. First, we focused on the differences between advertisements with and without metaphors. To obtain those differences, a *t*-test analysis was performed. The second stage was oriented to find differences in the complexity continuum. To get those results, we applied a repeated measures ANOVA. In the third stage, all the metrics (implicit and declarative) were regressed onto conceptual complexity derived from metaphorical content, with perceived complexity as a mediating variable. Finally, a Sobel test was used to statistically investigate the effect of the proposed mediator on the predictor–outcome relationship.

### Self-Reported Results

#### Perceived Complexity

The first step to analyze Hypothesis 1 was to validate that the complexity continuum was well constructed. For this purpose, a repeated measures ANOVA was performed. It showed that the perceived complexity of the levels of the ads was statistically and significantly different, *F*_(1.641,68.941)_ = 39.42, *p* = 0.000. More specifically, differences were found between juxtaposition (*M* = 6.1) and replacement (*M* = 4.9, *p* = 0.000) and between fusion (*M* = 5.9) and replacement (*M* = 4.9, *p* = 0.000).

**FIGURE 3:**
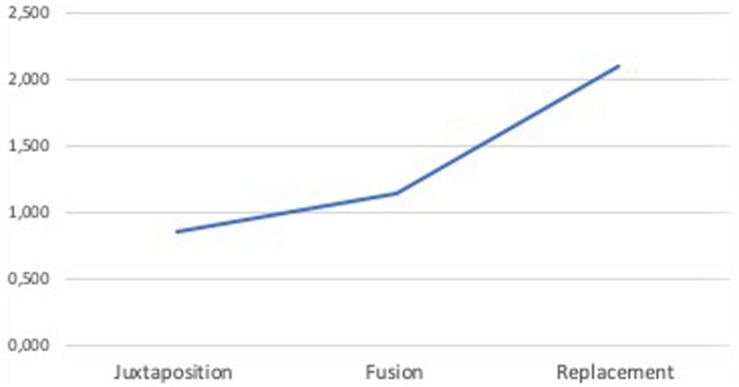
Perceived complexity continuum.

Note that in order to maintain the negativity of the scales to the left, subjects rated the images difficult to understand as 1 and those easy to understand as 7. Figure 3 shows a graphic re-interpretation to show that the complexity continuum was well perceived by the subjects.

To compare the perceived complexity between ads with and without metaphors, a paired-samples *t*-test was conducted. Results showed that there was a significant difference in the scores for ads without metaphors (*M* = 5.94, *SD* = 0.88) and ads including metaphors (*M* = 5.64 *SD* = 0.71); *t*(42) = −2.71, *p* = 0.010. It means that ads without metaphors were perceived as easier to understand than those with metaphorical content.

#### Attitude Toward the Advertisements

In order to obtain more accurate results of the self-reported measures, we performed two types of analysis: (1) a comparison between advertisements with and without metaphors and (2) a comparison between the three levels of complexity included in the experiment.

Regarding the analysis of Aad with and without metaphors, a paired-samples *t*-test was conducted. Results revealed a significant difference between advertisements without metaphors (*M* = 3.4, *SD* = 0.89) and those including metaphors (*M* = 4.5, *SD* = 0.67); *t*(42) = −9.235, *p* = 0.000.

**FIGURE 4:**
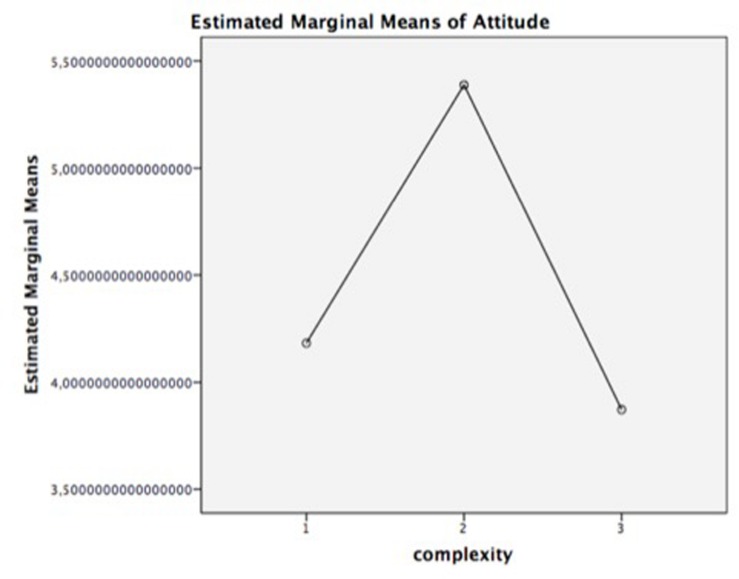
Mean of attitude toward the ad (Aad). Complexity: 1 = juxtaposition; 2 = fusion; 3 = replacement.

Subsequently, in order to test Hypothesis 1a, a repeated measures ANOVA was performed. It determined that Aad also showed statistically significant differences in the different complexity levels of the advertisement, *F*(2,84) = 75.877, *p* = 0.000. Regarding the level of complexity, *post hoc* tests using the Bonferroni correction revealed significant differences between juxtapositions (*M* = 4.8) and fusions (*M* = 5.4) and between fusions and replacement metaphors (*M* = 3.9). However, there were no significant differences between juxtaposition and replacement metaphors (see Figure 4).

**FIGURE 5:**
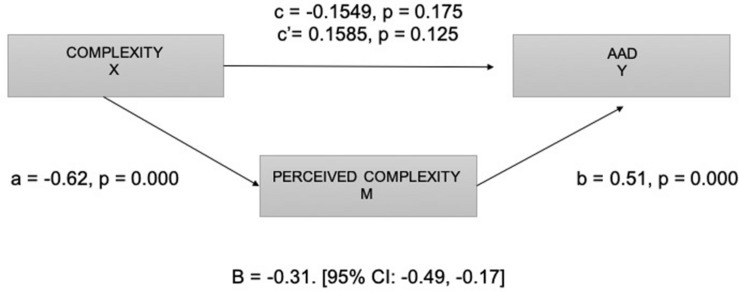
Diagram of the mediation model. Complexity (X), perceived complexity (M), and Aad (Y).

Finally, in order to validate the mediator effect of comprehension (perceived complexity) described as an inverted U-curve in the literature, a mediation analysis was performed. Statistically, mediation is often analyzed through path analytic models with one X variable, one mediator M, and one outcome variable Y (Papa et al., 2015). In the present study, the complexity of visual metaphors (X) is hypothesized to indirectly affect Aad (Y). In this model, higher levels of complexity are hypothesized to induce higher levels of perceived complexity, which in turn increase Aad. The indirect (or mediated) effect (B) is quantified as a × b and tested for statistical significance (see Figure 5).

The results of mediation analysis indicated that complexity of metaphors was a significant predictor of perceived complexity (*a* = −0.62, *p* = 0.000) and that perceived complexity was a significant predictor of Aad (*b* = 0.51, *p* = 0.000). These results support the mediational hypothesis. Ad complexity was not a significant predictor of Aad after controlling for the mediator (comprehension), *c*’ = 0.16, *p* = 0.179. The standardized indirect effect (B) was a (−0.62) × b (0.51) = −0.31 [95% CI: −0.49, −0.17].

A Sobel test was also conducted to validate the effect of the mediator, finding full mediation (*z* = −4.13, *p* = 0.000). The results revealed that comprehension/perceived complexity is a significant mediator of the relationship between advertisement complexity induced by metaphors and Aad; thus, the inverted U-curve pattern is validated, and consequently, results support Hypothesis 1a.

#### Purchase Intention

Regarding purchase intention, a paired-sample *t*-test revealed a significant difference between advertisements without metaphors (*M* = 3.7, *SD* = 0.91) and advertisements including metaphors (*M* = 4.0, *SD* = 0.84); *t*_(42)_ = −3.395, *p* = 0.020.

In order to test Hypothesis 1b, a repeated measures ANOVA also determined that the different complexity levels of the advertisement produced statistically significant differences in purchase intention, *F*_(2, 84)_ = 41.742, *p* = 0.000. *Post hoc* tests using the Bonferroni correction revealed significant differences between all three levels of complexity: juxtapositions (*M* = 4.8), fusions (*M* = 5.4), and replacement metaphors (*M* = 3.9).

**FIGURE 6:**
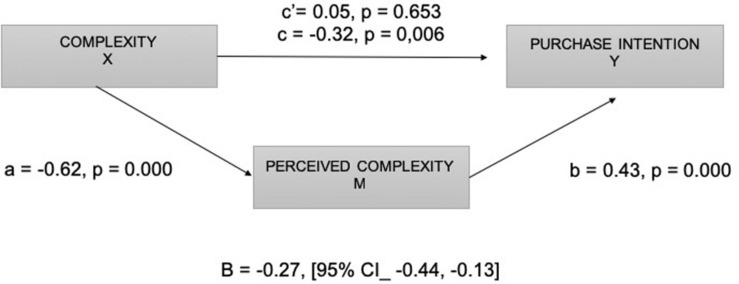
Diagram of the mediation model. Complexity (X), perceived complexity (M), and purchase intention (Y).

On the other hand, the mediation analysis indicated that advertisement complexity was a significant predictor of perceived complexity (*a* = −0.62, *p* = 0.000) and that perceived complexity was a significant predictor of purchase intention (*b* = 0.43, *p* = 0.000). Advertisement complexity induced by metaphors was no longer a significant predictor of purchase intention after controlling for the mediator (*c*’ = −0.05, *p* = 0.653), in consistence with a full mediation. The standardized indirect effect was a (−0.62) × b (0.43) = −0.27 [95% CI: −0.44, −0.13] (see Figure 6).

**FIGURE 7:**
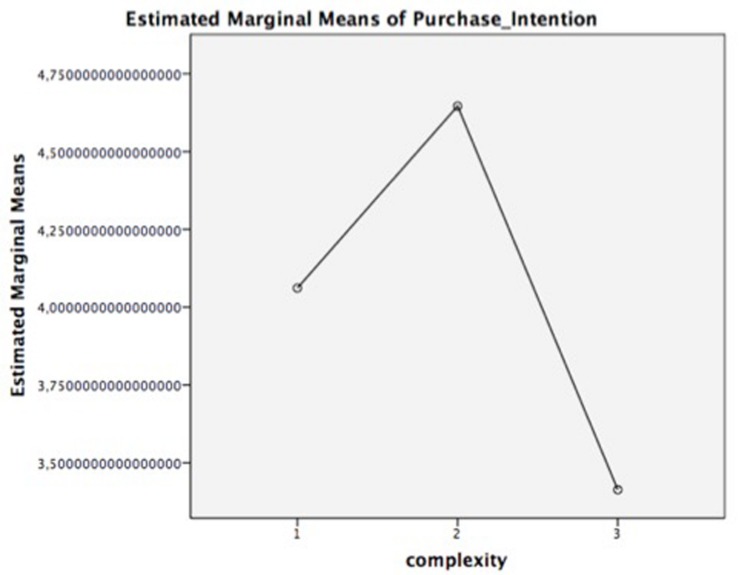
Mean of purchase intention. Complexity: 1 = juxtaposition; 2 = fusion; 3 = replacement.

Lastly, the Sobel test found full mediation (*z* = −3.64, *p* = 0.000). Thus, it can be concluded that comprehension mediated the relationship between advertisement complexity induced by metaphors and purchase intention. Consequently, the inverted U-curve pattern is validated for this construct, and the hypothesis 1b is supported, as displayed in Figure 7.

#### Preference

To test the preference hypothesis (H1c), due to the ordinal nature of the preference metric, a Friedman test was conducted as the non-parametric alternative to the one-way repeated measures ANOVA. The Friedman test revealed a statistically significant difference in preference depending on the complexity of the images visualized, χ^2^_(2)_ = 40.812, *p* = 0.000. A *post hoc* analysis with a Wilcoxon signed-rank test was conducted with a Bonferroni correction, resulting in a significance level set at *p* < 0.000. There were significant differences between the three levels of complexity.

**FIGURE 8:**
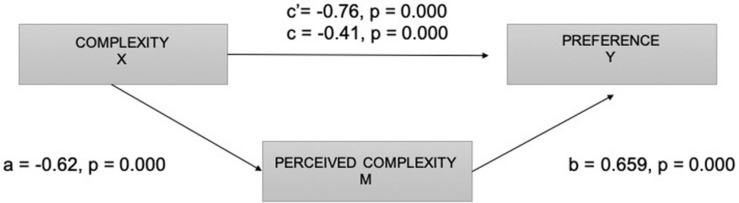
Diagram of the mediation model. Complexity (X), perceived complexity (M), and preference (Y).

To examine the effect of comprehension of different metaphors on preference, we performed a path analysis, described in Figure 8. Results indicated that complexity of visual metaphors was a significant predictor of comprehension (*a* = −0.062, *p* = 0.000). To investigate how conceptual complexity of metaphors could influence the preference of ads with metaphorical content, an ordinal logistic regression analysis was conducted. The conceptual complexity was found to contribute to the model [*X*^2^(1) = 15.053, *p* = 0.000]. The estimated odds ratio shows an inverse relationship between complexity and preference. It suggests a decreasing probability of improving the preference level with increasing complexity level of ads (complexity estimate = −0.760, SD = 0.205, Wald = 13.716, *p* = 0.000).

On the other hand, the analysis showed that comprehension was a significant predictor in the model [*X*^2^(1) = 16.536, *p* = 0.000]. The coefficient shows that when increasing the perceived complexity, there is a predicted increase of 0.659 in the log-odds of being in a higher level of preference (comprehension estimate = 0.659, SD = 0.1674, Wald = 15.103, *p* = 0.000). These results support the mediational hypothesis. The standardized indirect effect was a (−0.62) × b (0.659) = −0.4085.

Finally, a Wilcoxon signed-rank test was conducted to analyze the differences between ads with and without metaphorical content. Results showed statistically significant differences between (1) ads not including metaphorical images and ads including juxtapositions (*Z* = −4.059, *p* = 0.000) and (2) ads not including metaphorical images and fusions (*Z* = −4.908, *p* = 0.000). However, the analysis shows that there were no differences between ads including replacements and ads not including metaphorical images (*Z* = −0.775, *p* = 0.438).

Obtained results show that preference follows the inverted U-curve pattern according to which there is a positive relationship between complexity and positive feelings until a tipping point is reached where complexity exceeds comprehension. Thus, Hypothesis 1c is also supported. Table 3 shows a summary of the declarative results obtained.

**TABLE 3:** Summary of declarative results.

Measure	Differences between ads with metaphor and without metaphor	Differences among the three levels of complexity
Attitude toward the ad	*t*_(42)_ = −9.235, *p* = 0.000	*F*_(2.84)_ = 75.877, *p* = 0.000
Purchase intention	*t*_(42)_ = −3.395, *p* = 0.020	*F*_(2,84)_ = 41.742, *p* = 0.000
Preference	(1) *Z* = −4.059, *p* = 0.000 (2) *Z* = −4.908, *p* = 0.000 (3) *Z* = −0.775, *p* = 0.438	χ^2^_(2)_ = 40.812, *p* = 0.000

### Neurophysiological Results

#### Time in AOI

According to the literature, the total time spent exploring an image or an AOI (time in AOI) is a suitable indicator of comprehension (Raney et al., 2014) and cognitive load (Anderson et al., 2011) and is also a measure related to visual complexity (Henderson et al., 2003; Irwin, 2004). Therefore, in order to validate Hypothesis 2a, we analyzed the performance of time in AOI.

First, a paired-sample *t*-test was conducted to compare the time in AOI for ads including and not including metaphorical images. Results yielded a significant difference in the time in AOI between ads without metaphors (*M* = 4.8, *SD* = 0.81) and ads including metaphors (*M* = 4.5 *SD* = 0.62); *t*_(42)_ = −3.200, *p* = 0.003. Besides, a repeated measures ANOVA determined that the different complexity levels of the ads produced statistically significant differences in time in AOI, *F*_(1.38,57.96)_ = 11.609, *p* = 0.000. *Post hoc* tests using the Bonferroni correction revealed significant differences in time in AOI between juxtapositions (*M* = 4.1) and fusions (*M* = 4.8) and between fusions and replacements (*M* = 4.4).

**FIGURE 9:**
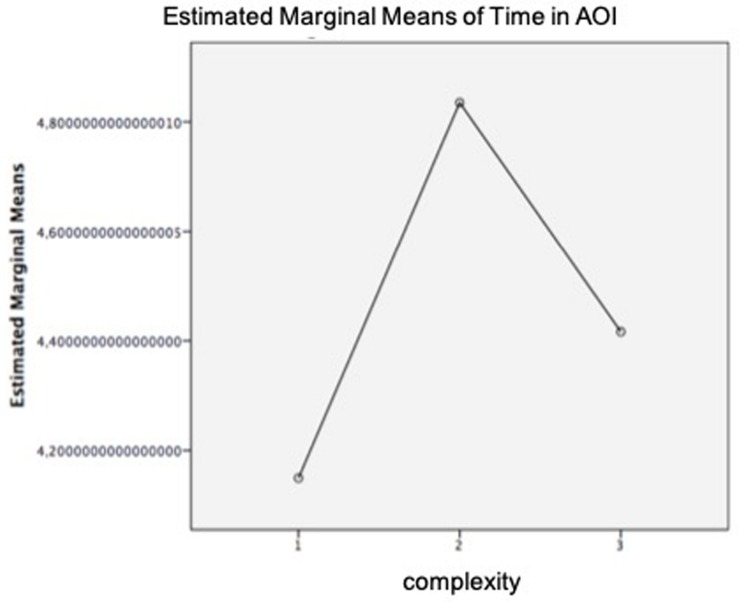
Mean of eye tracking (ET) time in area of interest (AOI). Complexity: 1 = juxtaposition; 2 = fusion; 3 = replacement.

As shown in Figure 9, time in AOI also follows the inverted U-curve pattern. Thus, to complete the analysis, a mediation was performed. However, the results obtained showed that, although ad complexity was a significant predictor of perceived complexity (*a* = 0.62, *p* = 0.000), perceived complexity was not a significant predictor of time in AOI (*b* = 0.07, *p* = 0.459), so perceived complexity did not mediate the relationship between ad complexity induced by metaphors and time in AOI (*B* = −0.043 [95% CI: −0.15, 0.05]). These results were also supported by results of the Sobel test (*z* = −0.74, *p* = 0.46).

Regarding the time spent exploring the ads, obtained results provide three interesting findings: (1) participants spent more time exploring ads without metaphors (*M* = 4.8) than ads with metaphors (*M* = 4.5); (2) the time that participants spent exploring an ad did not always increase when the ad increased in complexity (i.e., time in AOI fusion > time in AOI replacement); and (3) perceived complexity was not a significant predictor of time in AOI.

On the basis of the obtained results and since Hypothesis 2a predicted that higher levels of difficulty and, consequently, a higher index of cognitive load could be reflected by a longer time spent exploring the advertisement, we can state that Hypothesis 2a was not supported.

#### Cognitive Load Index

Cognitive load describes the relationship between the capacity of mental processing capability and the cognitive demands of a particular task (Mühl et al., 2014). Specifically, regarding the images, cognitive load is defined as the amount of resources needed to interpret a visualization (Anderson et al., 2011). As mentioned, the cognitive load should be reflected in physiological measurements and can be measured by various tools (García-Madariaga et al., 2019), but EEG has been proven the most reliable source of information of subjects’ cognitive load (Gevins et al., 1998; Antonenko et al., 2010).

In order to determine the cognitive load required by ads including and not including metaphorical images, a paired-sample *t*-test was conducted. Results revealed no significant difference in the scores for ads without metaphors (*M* = 27.1, *SD* = 8.32) and ads including metaphors (*M* = 27.6, *SD* = 6.84); *t*_(41)_ = −0.503, *p* = 0.617. However, a repeated measures ANOVA determined that cognitive load measured through EEG yielded statistically significant differences as a function of the different complexity levels of the ads, *F*_(2,82)_ = 3.102, *p* = 0.050. *Post hoc* tests using Bonferroni revealed differences in cognitive load between juxtaposition (*M* = 26.1) and replacement (*M* = 28.4).

**FIGURE 10:**
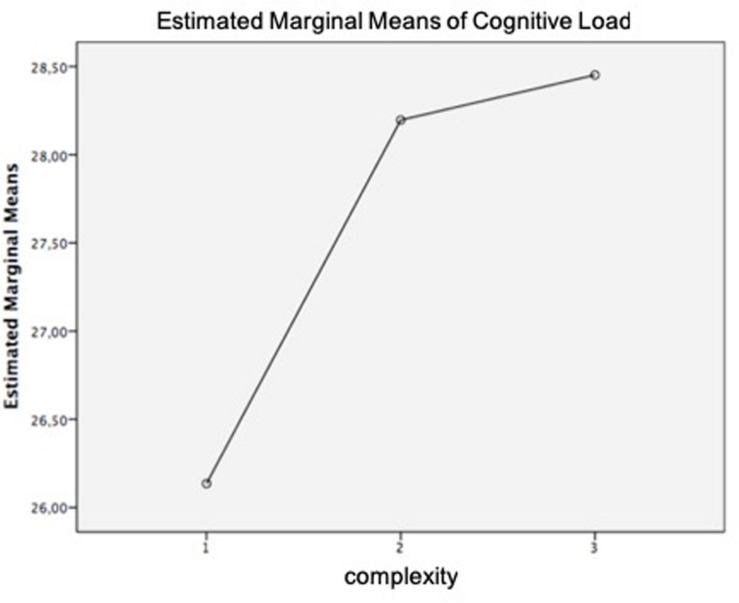
Mean of electroencephalogram (EEG) cognitive load. Complexity: 1 = juxtaposition; 2 = fusion; 3 = replacement.

Regarding these results, it is interesting to remark that EEG results reflect a linear relationship between complexity and cognitive load, as shown in Figure 10. This means that as complexity increases, so does cognitive load.

The mediation analysis showed that comprehension did not mediate the relationship between ad complexity induced by metaphorical content and cognitive load. Although ad complexity was a significant predictor of perceived complexity (*a* = −0.61, *p* = 0.000), perceived complexity was not a significant predictor of cognitive load (*b* = 0.31, *p* = 0.692), so perceived complexity did not mediate the relationship between ad complexity induced by metaphors and cognitive load (*B* = −0.18 [95% CI: −1.22, −0.74]. These results were also supported by the Sobel test performed (*z* = −0.39, *p* = 0.692).

According to the obtained results: (1) ads with metaphors had a higher cognitive load index (*M* = 27.6) than ads without metaphors (*M* = 27.1), (2) there is a linear relationship between complexity and cognitive load, and (3) perceived complexity is not a significant predictor of cognitive load. Since Hypothesis 2b stated that higher levels of difficulty could be reflected on a higher index of EEG cognitive load, we can state that Hypothesis 2a was supported.

#### Arousal

**FIGURE 11:**
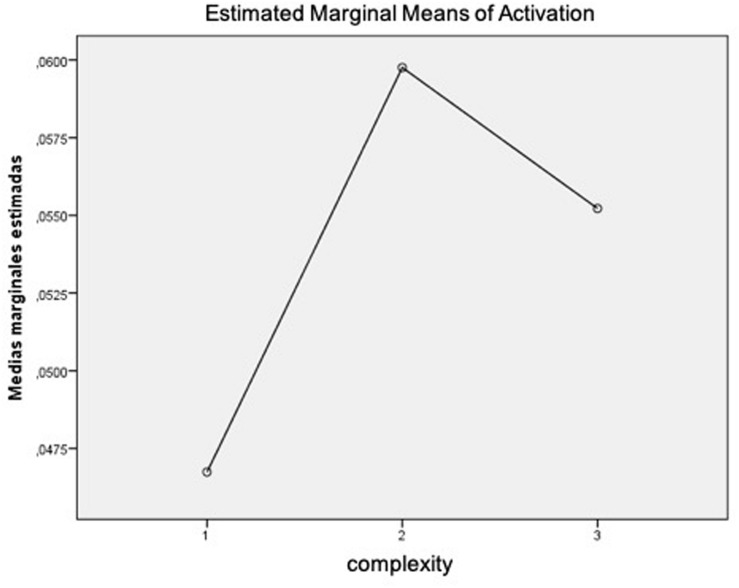
Mean of galvanic skin response (GSR) activation. Complexity: 1 = juxtaposition; 2 = fusion; 3 = replacement.

Again, the first step was to conduct a *t*-test to compare the activation induced by ads including and not including metaphorical images. Results revealed no significant differences in activation for ads without metaphors (*M* = 0.06, *SD* = 0.11) and ads including metaphors (*M* = 0.05, *SD* = 0.08); *t*_(38)_ = 0.372, *p* = 0.712. Nor was there any significant difference in activation as a function of the three levels of complexity, *F*_(1.20,45.62)_ = 0.288, *p* = 0.636. However, it is interesting to note that the means obtained reflect the aforementioned inverted U-curve pattern, as displayed in Figure 11.

Regarding the arousal, obtained results provide two findings: (1) participants had more arousal when they were exposed to ads without metaphors (*M* = 0.06) than when they visualized ads with metaphors (*M* = 0.05) and (2) the participants’ arousal did not always increase when the ad increased in complexity (i.e., time in AOI fusion > time in AOI replacement). On the basis of the obtained results and since Hypothesis 2c predicted that higher levels of difficulty and, consequently, a higher index of cognitive load could be reflected by higher levels of arousal, we can state that Hypothesis 2c was not supported. Table 4 shows a summary of the neurophysiological results obtained.

**TABLE 4:** Summary of neurophysiological results.

Measure	Differences between ads with metaphor and without metaphor	Differences among the three levels of complexity
Time in AOI (ET)	*t*_(42)_ = −3.200, *p* = 0.003	*F*_(1.38,57.96)_ = 11.609, *p* = 0.000
Cognitive load (EEG)	*t*_(41)_ = −0.503, *p* = 0.617	*F*_(2,82)_ = 3.102, *p* = 0.050
Activation (GSR)	*t*_(38)_ = 0.372, *p* = 0.712	*F*_(1.20,45.62)_ = 0.288, *p* = 0.636

## Discussion

According to Phillips (2003), the usage of metaphors in advertising has an impact on attention, elaboration, pleasure, and liking. As previous studies have proven the existence of an inverted U-curve pattern in advertisement appreciation (van Mulken et al., 2010, 2014), liking, and purchase intention (van Hooft et al., 2013), the aims of the present study were to validate previous results by testing this pattern in Aad, purchase intention, and preference, mainly because these measures are three important indicators of advertising effectiveness.

The obtained results validate previous results on purchase intentions in different product categories than used before (van Hooft et al., 2013) and provide new evidence related to preference and Aad by showing that these two metrics also follow the inverted U-curve pattern, according to which there is a positive relationship between complexity and positive feelings until a tipping point is reached where complexity exceeds comprehension (Phillips, 2000; McQuarrie and Mick, 2003; van Mulken et al., 2010, 2014; van Hooft et al., 2013). The fact that all the tested declarative constructs followed that pattern shows the importance of modulating the complexity of metaphors included in advertisements and of warning advertisers to be aware of including metaphorical content in advertisements because, instead of catching the consumers’ attention, this content could lead them to ignore the message and also the brand behind it.

Moreover, the results indicated that, regardless of metaphor type, ads with metaphors evoke more positive reactions than non-metaphor ads and that medium complexity is better to get more positive Aads, preference, and purchase intentions. This finding is consistent with van Mulken et al. (2010, 2014), who ensure that the impact of a message is maximized in the medium point of the complexity continuum, because the efforts demanded of consumers match those that they are willing and able to make available.

Regarding the neurophysiological techniques applied, the results of the present study are consistent to previous studies where it was proven that complex reasoning is almost invariably accompanied by activation of broad regions of the frontal and parietal cortices that form a frontoparietal network (Stamenković et al., 2019). Besides, we provide evidence of a linear relationship between complexity and cognitive load, consistent with the idea that increases in visual complexity increase the cognitive resources needed to elaborate the metaphorical content and provide a meaning for it (McQuarrie and Mick, 1999, 2003; Phillips, 2000; Jeong, 2008; Chang and Yen, 2013). As shown in the results, there were statistically significant differences in cognitive load index as a function of the different complexity levels of the ads. This demonstrated that as complexity increases, so does cognitive load.

On the other hand, although ET has been previously used to measure cognitive load (Pieters et al., 2010; Raney et al., 2014), results on time in AOI do not correlate with those obtained with EEG. Therefore, we cannot confirm the hypothesis that extra time is required to comprehend more complex metaphors. Consequently, we couldn’t confirm the hypothesis that the time spent exploring an ad is a reflection of the effort needed to comprehend it, because more complex metaphors did not necessarily require more fixation time to be understood. These findings are coincident with Wang et al. (2014) research according to which when an individual is conducting complex tasks, he/she is suffering high perceptual load, and if the load is too high, he/she might lose interest in exploring the stimuli and give up.

The same thing occurred with activation as measured by GSR. Despite that previous studies mention it as a useful tool to identify cognitive functions (Miller and Shmavonian, 1965; McEwen and Sapolsky, 1995; Shi et al., 2007; Mühl et al., 2014; Nourbakhsh et al., 2017, 2012), our experimental research results are not consistent with that idea. Instead, it turned out that our results were in line with Larmuseau et al. (2019) and Haapalainen et al. (2010), who did not obtain satisfactory results when attempting to detect cognitive load from GSR. Due to the fact that GSR is a convenient measure for indexing changes in sympathetic arousal that could be associated not only with cognition but also with emotion and attention (Vecchiato et al., 2014), our results may reflect the emotional meaning of visual metaphors (Venkatraman et al., 2015) or their attention-grabbing capacity (Critchley, 2002) instead of the cognitive load required to process them.

In this regard it is interesting that both, ET and GSR results followed the inverted U-curve pattern. This suggests that neither of the two methodologies is a suitable measures of cognitive load, at least, not in the specific case of visual metaphors. Instead, on the basis of our results, and taking into account that both metrics show the same behavior as the Aad and preference, it seems that time in AOI and arousal in this specific case are more closely related to the good feelings evoked by the ads.

## Conclusion

This study was motivated by the lack of evidence of consumers’ neurophysiological reactions to visual metaphors in advertising. In this regard, the results provide three conclusions. First, the EEG cognitive load index seems to be a suitable indicator of visual metaphors’ complexity, as it reflects the amount of cognitive resources needed to process them.

According to previous studies (Klimesch, 1999, 2012; Gevins and Smith, 2003; Anderson et al., 2011), suppression (decrease) of the alpha rhythm in the prefrontal cortex could be indicative of attentional demands, task difficulty, and cognitive load. The results of the present study provide evidence along the same lines, as we found a linear relationship between complexity and cognitive load, consistent with the idea that increases in visual complexity increase the cognitive resources needed to elaborate the metaphorical content and provide a meaning for it (McQuarrie and Mick, 1999, 2003; Phillips, 2000; Jeong, 2008; Chang and Yen, 2013).

Although further research is needed on this matter, the results on cognitive load represent a step forward for consumer neuroscience research, due to the fact that this measure could be refined and may be useful in the future to analyze consumers’ difficulty processing any kind of advertisement.

Second, according to the research, it turns out that both time spent exploring the stimuli and arousal follow the same “inverted U-curve” pattern as declarative constructs. This situation contradicts the previous findings that both metrics (Time in AOI and arousal) could be suitable to measure the cognitive load derived from complex tasks or contents (Miller and Shmavonian, 1965; Pieters et al., 2010; Raney et al., 2014; McEwen and Sapolsky, 1995; Shi et al., 2007; Mühl et al., 2014; Nourbakhsh et al., 2017, 2012). This conclusion supports the idea that neither of the two metrics is a suitable measure of cognitive load, at least, in the specific case of visual metaphors. Instead, in this specific context, they are more closely related to the good feelings evoked by the ads. Thus, practitioners must be careful when using GSR and ET in market research since regarding the evaluation of graphic ads, the metrics provided by those techniques are more related to attention and emotion than to cognitive load.

In advertising, visual metaphors are widely used to draw individuals’ attention and entice them to buy the product (van Mulken, 2003). However, their usage is not simple. Based on the findings of the present study, researchers should be aware of the importance of comprehension in consumers’ reactions. Practitioners should take care to modulate the complexity of metaphors and to prevent a lack of comprehension from reducing the effectiveness of these rhetorical figures. In any case, the study of visual metaphors in advertising should continue because only by going deeper into the understanding of consumer reactions to these resources will marketers be able to take advantage of them.

Visual metaphors are creative resources that try to provide differentiation and recognition of the brand. Our results highlight the importance of assessing the perceived complexity of ads in order to find the optimal level of complexity of the visual metaphors to achieve the desired brand awareness and advertising effectiveness (van Mulken et al., 2014; Pilelienė and Grigaliūnaitė, 2016).

Finally, the present study provides evidence on how useful the use of neuroscientific techniques can be to have an objective measure of consumer reactions to marketing stimuli and to find new and useful insights that cannot be detected with declarative methodologies (García-Madariaga et al., 2019). Our results suggest that EEG seems to be not only an adequate technique to discover how advertising processing can be when visual metaphors are included but also a suitable indicator of complexity, as it reflects the amount of cognitive resources needed to process stimuli. Although further research is required to deepen this issue and test the measure of cognitive load in other types of advertising and contexts, current evidence represents a step forward for consumer neuroscience research.

## Limitations and Further Research

While this study provides theoretical and practical implications, some limitations must be acknowledged, mainly regarding the sample size, similarity of participants, and forced exposure to the stimuli.

Although previous studies have demonstrated that consumer neuroscience studies with small sample sizes can produce predictive results and significant insights (Berns and Moore, 2012; Smidts et al., 2014; Shen and Morris, 2016; Christoforou et al., 2017), the use of larger samples could broaden the generalizability of the findings.

Besides, the sample used is very similar because it is formed by students, and their age, education level, cultural context, and lifestyle are pretty similar. Since previous studies have proven significant cross-cultural variations in the interpretation of advertisements (Margariti et al., 2019), it would be interesting to vary the sample and to analyze how variables such as education, culture, and environment influence the processing of metaphorical content included in ads and how those variables could impact on the four variables mentioned by Phillips (2003): attention, elaboration, pleasure, and liking.

On the other hand, the present study was conducted in a laboratory, and subjects were forced to visualize the stimuli contrary to how they behave in real life, where they devote their attention voluntarily. The degree of control exerted over potential extraneous variables determines the level of internal validity of a study (Slack and Draugalis, 2001). Although the laboratory context provides that control and consequently increases the internal validity of the study, it would be interesting to replicate the present study in more real conditions and analyze if the processing of metaphorical content varies when visualization is not forced.

Finally, on the basis of previous findings according to which advertisement appreciation follows an inverted U-curve pattern (Phillips, 2000; McQuarrie and Mick, 2003), we took comprehension as a mediation variable. Future research could consider other mediating variables such as attitude toward the brand, product category, education level, or cultural context.

## Data Availability Statement

The datasets generated for this study are available on request to the corresponding author.

## Ethics Statement

Ethical review and approval was not required for the study on human participants in accordance with the local legislation and institutional requirements. The patients/participants provided their written informed consent to participate in this study.

## Author Contributions

All authors listed contributed to the design of the experiment, data collection, data analysis, literature review and writing and reviewing of this manuscript.

## Conflict of Interest

The authors declare that the research was conducted in the absence of any commercial or financial relationships that could be construed as a potential conflict of interest.
